# Major Depressive Episode and Generalized Anxiety Disorder Among Sexual Minority Adults in the United States

**DOI:** 10.1093/geroni/igaa057.3172

**Published:** 2020-12-16

**Authors:** Cara Exten, Anna Salomaa

**Affiliations:** Penn State University, University Park, Pennsylvania, United States

## Abstract

Sexual minority (SM) health disparities constitute a serious public health concern, and disparities in mental health are among the most striking. Among a nationally-representative sample (NESARC-III) of US adults (aged 18-66), we used time-varying effects models (TVEM) to estimate the age-varying prevalences of past year major depressive episode (pyMDE) and generalized anxiety disorder (pyGAD) by SM status and biological sex. pyMDE and pyGAD were most common among SM women, followed by SM men, heterosexual women, and heterosexual men. pyMDE was highest among SM women and SM men in early adulthood with a second peak in the mid-50s (women)and around age 40 (men). pyGAD was highest among SM women aged 54-60 and among SM men aged 30-33. Our findings reveal that older adulthood may be a time of increased risk for pyMDE and pyGAD among SM women. Future work should explore factors that contribute to this increased risk.

